# Update on the therapy of adult-onset Still’s disease with a focus on IL-1-inhibition: a systematic review

**DOI:** 10.1177/1759720X211059598

**Published:** 2021-11-24

**Authors:** Claudia Kedor, Stylianos Tomaras, Daniel Baeumer, Eugen Feist

**Affiliations:** Corporate Member, Department of Rheumatology and Clinical Immunology and Berlin Institute of Health, Institute of Medical Immunology, Charité – Universitätsmedizin Berlin, Freie Universität Berlin, Humboldt Universität zu Berlin, 10117 Berlin, Germany; Department of Rheumatology, Helios Clinic Vogelsang-Gommern, Gommern, Germany; Novartis Pharma GmbH, Nürnberg, Germany; Department of Rheumatology, Helios Clinic Vogelsang-Gommern, Gommern, Germany

**Keywords:** adult-onset Still’s disease, anakinra, canakinumab, IL-1 inhibition, tadekinig, treatment

## Abstract

**Introduction:**

The past decade has seen increasingly rapid advances in understanding the pathogenic nature of adult-onset Still’s disease (AOSD) and its shared symptoms with the systemic juvenile idiopathic arthritis (sJIA). Interleukin-1 (IL-1) blocking agents are key elements in the treatment. In this updated systematic review, we focus on studies on efficacy and safety of IL-1 blockers published in the past 5 years and review on latest available therapies.

**Methods:**

We conducted searches using Medline, Biosis, Embase, and Cochrane databases between 2016 and 2021 using the terms AOSD, IL1, IL-18, canakinumab, anakinra, tadekinig, and rilonacept and if applicable their trade names. Duplicates, case reports, and manuscripts with incomplete data were excluded.

**Results:**

Of the 1013 screened publications, 17 were eligible after careful selection. We only found two published randomized controlled studies in the past 5 years. Review manuscripts of rare diseases, like our work, usually rely on retrospective studies and case series. Anakinra and canakinumab can be successfully used as first- or further-line treatment in patients with AOSD refractory to steroids. A homogeneous outcome is not established yet. Thus, a combination of clinical and laboratory tests can support the experienced clinician in the decision-making process.

**Conclusion:**

The approval of IL-1 inhibitors for AOSD brought us into a new era in the treatment of AOSD. The overall efficacy-safety profile of the IL-1 inhibitors is favorable reflecting a targeted approach as standard of care. We can expect that the successful treatment of AOSD with IL-1 inhibition will facilitate further clinical and basic research with impact on other auto-inflammatory and hyper-inflammatory conditions.

In this systematic review, we analyzed papers published in the previous five years (2016-2021) with a focus on IL-1 inhibitors, the only class of biologic drugs approved for therapy of adult-onset Still’s disease. We also discussed the latest clinical trials and therapeutic approaches for new drug candidates, such as Tadekinig.

## Introduction

Adult-onset Still’s disease (AOSD) represents one of the multifactorial fever syndromes for which a major breakthrough has been achieved in the past years. At first, a uniform consent on clinical grounds and biomarker profiling was obtained between pediatric and adult rheumatologist for the concept of a disease continuum of systemic juvenile idiopathic arthritis (sJIA) and AOSD, which are separated just by age of 16 for rather formal reasons.^1^ This led to a tight cooperation with discussions on common diagnostic criteria, tools for monitoring of disease activity, as well as treatment strategies and goals.

An especially fruitful exchange is still ongoing with respect to improvement of treatment options. In this context, initial experiences with Interleukin-1 (IL-1) blocking agents in patients with AOSD led to first controlled clinical studies in sJIA with approval of canakinumab as well as anakinra. Subsequently, accumulating evidence from retrospective and prospective cohort studies as well as small investigator-initiated controlled clinical studies also led to an approval of the same compounds for AOSD. In this context, the regulatory authorities paid also attention to and agreed on the concept of a disease continuum.

The IL-1 signaling pathway was identified to play a central role in the pathogenesis of sJIA as well as AOSD by several *in vitro* studies.^2^ In correlation with disease activity, most of the identified biomarkers attribute to an IL-1 signature. In this systematic review, we compile and discuss the published literature on efficacy and safety of IL-1 blocking agents and the closely related target IL-18 in the treatment of AOSD over the past 5 years.

## Methods

**Figure 1.**
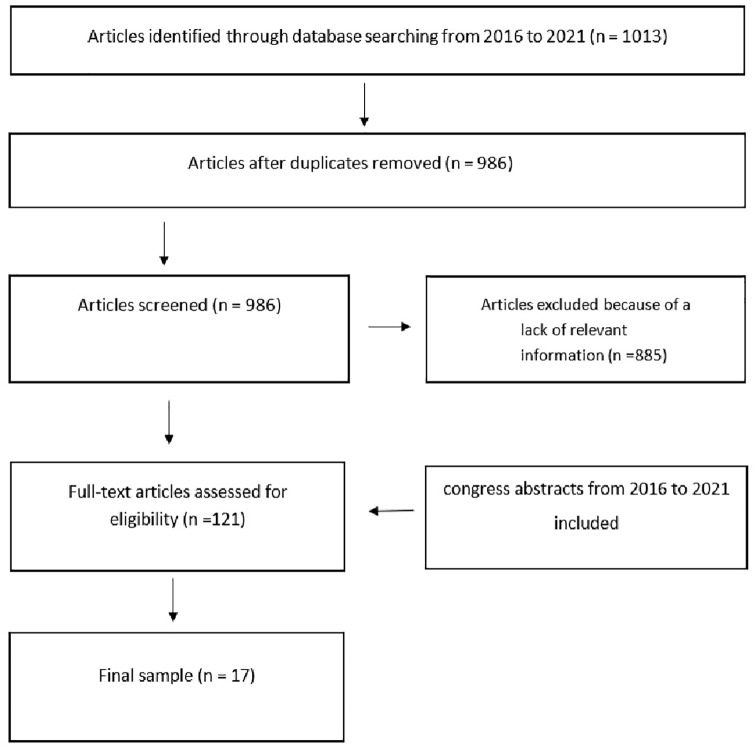
PRISMA flow diagram of record selection process: canakinumab, anakinra, rilonacept results, Medline, Embase, Biosis, Cochrane, abstracts (search conducted on 8 March 2021).

In 2015, Jamilloux *et al.*^3^ published an important review of treatment of AOSD. So, we conducted searches using Medline, Biosis, Embase, and Cochrane databases between 2016 and 2021 using the terms: AOSD, IL1, IL-18, canakinumab, anakinra, tadekinig, rilonacept, and IRAK inhibitors, and if applicable their trade names (Supplemental Material). Duplicates, case reports, reviews, and manuscripts with incomplete data have been excluded. We identified 1013 publications, 27 duplicates were eliminated, and after screening for period (2016–2021) and content, 121 were left to consider. Eligible were 17 (Figure 1). We did not include IRAK-inhibitors in the final draft, because of lack of data.

## Efficacy

We only found two published randomized controlled studies in the past 5 years. We excluded review manuscripts of rare diseases, like our work, usually rely on retrospective studies and case series. Table 1 summarizes the results.

**Table 1.** Overview of the use of IL-1 inhibitors in AOSD, including treatment response and effect on steroid use.

Primary author	Year	Drug	Study design	Patients on treatment (n)	Gender (M/F)	Previous treatment	Systemic AOSD	Arthritic AOSD	Duration of treatment (months)	Reasons for withdrawal	Degree of remission			Steroid use		
											Complete	Partial	None	n	Stopped	Reduced
Laskari *et al.*^4^	2020	CAN	M, R	39	n.a.	c, s, and bDMARDs	n.a.	67%	12	n.a.	68%^a^	16%^a^	16%^b^	41	21 (weaned)	n.a.
Vitale *et al.*^5^	2020	CAN	M, R	9	1/8	NSAIDs, GC, MTX, HCQ, CyA, ANA, TCZ, ADA, ETN	7	2	15 ± 12.3	n.a.	8	n.a.	1	9	3	3
^c^Tomelleri *et al.*^6^	2020	CAN	SC, R	13	8/5	ANA, TCZ, TNF-Inh, cDMARD	n.a.	n.a.	3–18	Disease control	13	0	0	13	3	10
Cavalli *et al.*^7^	2019	CAN	R	4	n.m.	GC, MTX	4	n.a.	n.a.	Complete response	4	n.a.	n.a.	4	2	n.a.
^d^Colafrancesco *et al.*^8^	2017	CAN	R	4	n.m.	MTX, HCQ, CyA, IFX, ETN, ADA, TCZ	2	2	22.1 ± 6.5	Remission (1), loss of efficacy (1)	3	0	1	4	0	3
^c^Ugurlu *et al.*^9^	2018	CAN	R	10	2/8	MTX, LEF, TCZ, ANA, IFX, ADA, ETN, RTX	n.a.	n.a.	43 ± 33	TBC (1), disease control (1), n.m. (1)	10	0	0	10	4	n.a.
Vitale *et al.*^10^	2016	CAN	R	9	1/8	GC, NSAIDs, cDMARDs, ANA, TCZ, ADA, ETN	n.a.	n.a.	n.a.	Lack of efficacy (1)	2/3 (66.66%)	1/3 (33.33%)	0/3 (0%)	n.a.	n.a.	n.a.
Kedor *et al.*^11^	2020	CAN	RCT	18	8/10	GC, NSAIDs, ANA, TCZ, TNF-Inh	0	18	24	Non-response (5)	10	8	0	Stable dose	⩽10 mg/day PND	
Bodard *et al.*^12^	2021	ANA	M, R	23			n.a.	n.a.	n.a.		n.a.	n.a.	n.a.	n.a.	n.a.	n.a.
Campochiaro *et al.*^13^	2021	ANA	SC, R	41			25 (61%)	16 (39%)	24		20 (49%)	5 (24%)	3 (7%)	41 (23 ± 18) PND	38% (EOT)	n.a.
^c^Schanberg *et al.*^14^	2020	ANA	RCT	6	n.m.	n.a.	n.a.	n.a.	0.5	n.a.	6 ACR30 response	0	0	n.a.	n.a.	n.a
Colafrancesco *et al.*^8^	2017	ANA	M, R	140	47/93	GC, NSAIDs, cDMARDs, IFX, ETN	104 (74.2%)	36 (25.8%)	35.7 ± 36.1	Remission, AE	20 (28.1%)	n.a.	n.a.	97.8%	55.6% (month 12)	n.a.
Sfriso *et al.*^15^	2016	ANA	M, R	35	n.m.	GC, NSAIDs, TNF-Inh,	n.a.		n.a.	Remission, AE, loss or lack of efficacy	26	7	1	84.5%	n.a.	n.a.
Vitale *et al.*^10^	2016	ANA	M, R	78	n.m.	GC, NSAIDs, cDMARDs, biologics	n.a.		n.a.	n.m.	61/78 (78.2%)	10/78 (12.82%)	7/78 (8.97%)	n.a.	n.a.	n.a.
Vitale *et al.*^5^	2020	ANA	M, R	141	48/93	GC, NSAIDs, cDMARDs, biologics	105 (74.5%)	36 (25.5%)	12	GC, NSAIDs, cDMARDs, biologics	n.a.	n.a.	n.a.	n.a.	n.a.	n.a.
Dall’Ara *et al.*^16^	2016	ANA	M, R	13	4/9	GC, NSAIDs, IVIG, cDMARDs	8	5	12–102	Remission, AE, non-compliance, LTFU	13	1	n.a.	6	n.a.	n.a.
Gao and Petryna^17^	2016	RIL	R, SC	2	1/1	n.m.	n.a.	n.a.	3	n.m.	Good response	n.a.	n.a.	n.a.	n.a.	n.a.

ACR, American College of Rheumatology; ADA, adalimumab; AE, adverse event; ANA, anakinra; AOSD, adult-onset Still’s disease; b, biologic; CAN, canakinumab; cDMARDs, conventional disease modifying antirheumatic drugs; CyA, cyclosporine A; EOT, at the end of treatment; ETN, etanercept; GC, glucocorticosteroid; HCQ, hydroxychloroquine; IFX, infliximab; IL, interleukin; IVIG, intravenous immunoglobulin; LEF, leflunomide; LTFU, lost to follow-up; M, multicenter; MTX, methotrexate; n.a., not applicable, nor available; n.m., not mentioned; NSAIDs, non-steroidal anti-inflammatory drugs; PND, prednisone; R, retrospective; RCT, randomized controlled trial; RIL, rilonacept; RTX, rituximab; s, synthetic; SC, single center; TBC, to be confirmed; TCZ, tocilizumab; TNF-Inh, tumor necrosis factor inhibitor.

a All patients at last visits (n = 37).

b Patients with relapse.

c Congress abstract.

d All patients switched from ANA to CAN because of inefficacy (n = 1 discontinued).

### Anakinra

Anakinra was the first biologic drug introduced that binds directly to IL-1 receptors and differs from naturally occurring IL-1 by the presence of a methionine group. We found 10 papers published in the previous 5 years (2016–2021) involving a total of 479 patients with AOSD treated with anakinra.^1^ An important systematic review about treatment with anakinra in AOSD has been published in 2018.^18^

Bodard *et al.*^12^ investigated the efficacy of anakinra in 23 patients with AOSD, of whom eight had cardiac involvement including pericarditis and myocarditis with tamponade, and reported positive results in all of them. Campochiaro *et al.*^13^ found drug retention rates (DRR) of 53.1% after 24 months treatment with anakinra in 41 patients with AOSD. In a randomized, placebo-controlled study by Schanberg *et al.*^14^ assessing the use of anakinra in 12 patients with Still’s disease (nine children and three adults, n = 6 placebo, n = 6 anakinra), 6 patients on anakinra showed rapid response at week 2, defined as absence of fever and 30% improvement of American College of Rheumatology criteria (ACR30).

Colanfrancesco *et al.* conducted a large study with a total of 140 patients with AOSD from 18 different centers in Italy. The mean disease duration was 50.3 months ± 81.67 and the majority of participants (n = 111) received anakinra as first-line biologic agent. Commonly reported previous treatments were non-steroidal anti-inflammatory drugs (NSAIDs), glucocorticoids, or other disease-modifying antirheumatic drugs (DMARDs). Results showed a significant improvement of clinical and serological disease parameters within the first 3 months of treatment. Primary and secondary lack of efficacy after 12 months of treatment was reported in 15/140 and 11/140 patients, respectively.^8^

Ruscitti *et al.* published a paper in which they mention six patients with AOSD treated with anakinra and/or conventional DMARDs and/or steroids. This study was not designed to analyze drug efficacy.^19^ Sfriso *et al.* studied the effect of anakinra as first- or second-line biologic treatment on 35 Italian patients with AOSD. About 3 years after initiation, 21 of 35 were still on treatment.^15^

Vitale *et al.* carried out a number of investigations in a multicentre study with 141 patients with AOSD in Italy in order to compare early *versus* delayed anakinra treatment. Participants had a mean disease duration of 50.4 months and were retrospectively assigned to different treatment groups depending on duration of disease, duration of treatment with anakinra, interval between disease onset and treatment initiation, and previous treatments strategies (mainly including glucocorticoids, DMARDs, and other biologic agents). Drug efficacy was measured by clinical, serological manifestations and Pouchot’s activity score. There were no statistically significant differences in efficacy between groups after 3, 6, and 12 months. However, there may be a faster control of systemic inflammation and articular manifestations in patients receiving anakinra as soon as after AOSD onset. The DRR was 44.6% and 30.5% in a 60- and 120-month period, respectively.^5^ According to another analysis of 78 patients treated with anakinra for AOSD by Vitale *et al.*,^10^ 78.2% had a complete response, 12.8% a partial response, and only 9% were non-responders.

In a case series by Dall’Ara *et al.*,^16^ 13 patients with AOSD were treated with anakinra as first- or second-line biologic therapy, of whom 12 showed complete remission during a median follow-up time of 61 months. In a case report by Chalasani *et al.*,^20^ one 21-year-old male patient, who was refractory to high-dose glucocorticoids and 100 mg anakinra/day, was treated with 200 mg/day in combination with methotrexate. In their review of 15 patients treated with anakinra, Vercruysse *et al.*^21^ concluded that there are two main factors associated with a substantial treatment response: a systemic form and the absence of arthritis.

Kougkas *et al.* published results of a single-center retrospective study of patients with AOSD distinguishing two phenotypes: the chronic articular and the systemic one. Patients with a systemic subtype were receiving anakinra (n = 4), tocilizumab (n = 3), adalimumab (n = 1), etanercept (n = 1), and canakinumab (n = 1). At the end of follow-up period, six patients were still receiving the initial treatment.^22^ Yazici *et al.* demonstrated their results of the TURKBIO biological registry. The second most frequently used biological treatment in patients with AOSD after tocilizumab (n = 13) was anakinra (n = 4). However, results about treatment efficacy were not published as of April 2021.^23^ Marketos *et al.* reported a case report of a 79-year-old man with arthritis, skin rash, and pleural effusion, who was treated successfully with anakinra. This elderly patient with AOSD could reach remission after 7 days, while on background combination with steroids.^24^ In their analysis on DRR in adult and pediatric patients with Still’s disease treated with anakinra, Sota *et al.* showed rates of 74.3%, 62.9%, 49.4%, and 49.4% after 12, 24, 48, and 60 months of follow-up, respectively. There were no significant differences between adults and children. Nor did the co-administration of conventional DMARDs influence the outcome.^25^

### Canakinumab

Canakinumab is a long-acting high-affinity, fully human-IL-1ß monoclonal antibody that belongs to the IgG1/k isotype subclass. Canakinumab specifically blocks the interaction between IL-1ß and IL-receptors and leads to inhibition of downstream targets, actions that prevent the production of inflammatory mediators.^26^ Canakinumab has a half-life of 26 days in pediatric population. We found nine papers published in the previous 5 years (2016–2021) involving a total of 110 patients with AOSD treated with canakinumab (Table 1).

Ugurlu *et al.* published a paper in which 11 patients with therapy refractory AOSD treated with canakinumab. Previous treatments include corticosteroids, conventional DMARDs (methotrexate, leflunomide), and biologic DMARDs (tocilizumab, anakinra, infliximab, adalimumab, etanercept, and rituximab). The timespan between initial diagnosis and initiation of canakinumab treatment was 43.2 ± 28 months and the mean number of injections was 11.8 ± 6. The mean follow-up period was 42.2 ± 31 months. Eight out of 11 patients were still receiving canakinumab 300 mg/month, 150 mg/month, or 150 mg every 2 weeks. One patient achieved full remission after one single injection. Patient-reported global visual analogue scale (VAS), ferritin, and erythrocyte sedimentation rate (ESR) have significantly improved. At the time of analysis, six patients were on background corticosteroids up to 10 mg prednisolone/day maximum.^9^ Colafrancesco *et al.* identified four patients with AOSD, who switched from anakinra to canakinumab. After a mean duration of 22.1 months, the results were promising. One patient achieved remission after 45 months and stopped therapy without relapsing during follow-up. Two patients did respond to canakinumab and continued treatment, and in one patient, therapy proved to be ineffective.^8^

In their case series, Cavalli *et al.* examined the efficacy of canakinumab (4 mg/kg/4 weeks) as first-line biologic agent in four patients with AOSD, refractory to corticosteroids, and methotrexate. Canakinumab markedly improved clinical features as well as laboratory parameters in all patients. Efficacy was evaluated by analyzing fever, skin rash, arthritis, pericarditis, hepatosplenomegaly, CRP, ESR, and serum ferritin. The strong anti-inflammatory effect of canakinumab in this small case series led to a significant steroid-sparing effect.^7^ Similarly, Campochiaro *et al.* reported substantial response in 6 of 10 patients with AOSD after treatment with canakinumab for a median duration of 36.5 months. The median follow-up ranged between 4 and 18 months with a median of nine. Previous treatments including corticosteroids, conventional DMARDs, and anakinra (n = 5) were ineffective. Regardless of prior therapy regimens, canakinumab in a dose of 300 mg every 4 weeks led to a rapid resolution of clinical and laboratory signs of disease activity. Furthermore, concomitant use of steroids and DMARDs were tapered or even discontinued without relapses.^27^

Laskari *et al.* assessed the efficacy of canakinumab in 39 patients with refractory AOSD and 11 children with a systemic form of juvenile idiopathic arthritis. Prior treatments with corticosteroids, cDMARDs, bDMARDs, and IVIGs proved to be insufficient to achieve remission. Patients received canakinumab in a dose of 4 mg/kg/4 weeks (maximum 300 mg), 150 mg/month, or 150 mg/2 weeks. Disease activity was defined as the presence of at least one of systemic symptoms (fever, rash, lymphadenopathy, hepatosplenomegaly, serositis, sore throat) and/or arthralgias/arthritis and at least one abnormal laboratory marker (hematological profile, ESR, CRP, ferritin, liver enzymes). Clinically and serologically, success was observed during the first month of treatment and could be measured by using the Pouchot scoring system. Serositis improved significantly within the first 3 months of treatment. This study demonstrated that at 3 months 22 patients (44%) showed a complete response and nine (18%) a partial response. An overall complete response at last visit after 12 months was observed in 60% of patients, while 18% only showed partial response. Persisting activity at last visit consisted mainly of arthralgias/arthritis, and elevated CRP, less frequently of low-grade fever, lymphadenopathy, serositis, rash, increased white cell count, anemia, elevated ferritin, ESR, and platelet count. Secondary outcome of the same study was to evaluate whether canakinumab could be tapered in patients in remission. After increasing the interval in between injections, one out of 15 patients did relapse after 8 months. The higher relapse rate occurred after canakinumab discontinuation. Relapse management based on re-introduction or intensification of canakinumab was successful.^4^

Vitale *et al.* provided analysis of nine patients with AOSD treated with canakinumab in a dose of 150 mg every 4 weeks. The median disease duration was 15 months. Patients had been treated previously with corticosteroids, NSAIDs, cDMARDs (methotrexate, leflunomid, cyclosporine A) and biologic agents (tocilizumab, adalimumab, etanercept, and in four cases with anakinra). Four patients received canakinumab as monotherapy. Persisting signs or symptoms within the first 3 months led to a dose increase to 300 mg every 4 weeks in two patients. The majority of the patients (eight out of nine) achieved remission at 3 months. In one patient, canakinumab had to be stopped at month 6 because of inefficacy with fever episodes, myalgia, arthritis, and leucocytosis. A long-term remission after 45 months led to treatment cessation in one case. Overall, there was a significant reduction in corticosteroids at month 3 in this study. Two patients were even able to stop background steroids while on canakinumab. The same with concomitant methotrexate therapy in two cases.^5^ In a smaller case series, Vitale *et al.*^10^ found a complete response in two out of three patients treated with canakinumab for AOSD. The remaining patient experienced a partial response.

Kedor *et al.* examined the efficacy of canakinumab in the treatment of refractory AOSD with articular manifestation in a randomized, double-blind, placebo-controlled, multicenter trial. Endpoints of the study were a clinically relevant reduction in articular manifestation, fever episodes, patient-reported outcomes, and ACR response criteria for rheumatoid arthritis. Eighteen patients received canakinumab (4 mg/kg/4 weeks, maximum 300 mg) and 17 received placebo in the first 3 months. After that period, non-responders on placebo switched to canakinumab, as rescue therapy. Responders received open-label medication after 6 months of blinded period. Twelve patients had a reduction in DAS28 (ESR) of more than 1.2 after the 12-week period. At week 12, 77.8% and 64.7% of patients were free from fever in the canakinumab and in the placebo-arm, respectively. Skin manifestations were similar in both groups after 12 weeks. Non-responders on placebo had a similar outcome after receiving canakinumab as those in the verum group at baseline. Ten out of 12 canakinumab responders at week 12 remained responders until week 24, while two remained responders until week 20. Seven patients entered the long-term extension period: four patients remained in DAS28 remission and three in low-disease activity.^11^

Tomerelli *et al.* reported 13 AOSD patients treated with canakinumab for a follow-up ranging from 3 to 18 months showing a striking and rapid clinical response and a significant steroid-sparing effect.^6^ Kougkas *et al.* published results of a single-center retrospective study of patients with AOSD distinguishing two phenotypes in AOSD: the chronic articular and the systemic one. Canakinumab was used in 1 of 10 patients with systemic form. Other medications included anakinra (n = 4), tocilizumab (n = 3), adalimumab (n = 1), and etanercept (n = 1). At the end of follow-up period, six patients were still receiving initial biologic treatment.^22^

### Rilonacept

There exist limited data to guide treatment decisions to start rilonacept, a long-acting IL-1 receptor fusion protein consisting of the Fc portion of human IgG1 and the human IL-1 receptor, in patients with AOSD. Gao and Petryna^17^ reported two cases of successful treatment of refractory patients with AOSD, despite the use of prednisone, methotrexate, and anakinra.

### Tadekinig

Gabay *et al.* investigated the effect of tadekinig alfa, a recombinant human interleukin-18 binding protein in an open-label, multicenter, dose escalating phase II clinical trial. Twenty-three patients with active AOSD despite treatment with prednisone, conventional or even biological DMARDs were treated for 12 weeks with either 80 mg or 160 mg, or even with 320 mg in case of not achieving predicted response criteria after 3 weeks. Tadekinig alfa was given three times a week subcutaneously. The primary endpoint of this study was powered for safety, nevertheless 11 patients achieved the response criteria defined as a 50% or greater reduction of CRP values compared with baseline, and resolution of fever. At week 12, 7 of 13 patients with skin rash at baseline showed improvement. The levels of ferritin, IL-6, neutrophils, S100A8/9, and S100A12 were significantly decreased. Interestingly, all responders with elevated IL-18 at baseline had undetectable free IL-18 at final blood assessment.^28^

Kiltz *et al.* published a paper, in which two patients with AOSD were treated with Tadekinig alfa for several months. The first patient sustained clinical remission for 2 years, while on background prednisone below 5 mg/day. The second patient received Tadekinig alfa for more than 2 years with maintained clinical response.^29^

### New drugs

#### AEVI-007

AEVI-007 is a fully human anti-IL-18 monoclonal antibody and is being evaluated since March 2021 in a phase 1b multicentre, open-label study to evaluate safety, tolerability, efficacy, pharmacokinetics, and pharmacodynamics in patients with AOSD. Trial planning includes 12 patients (clinical trials.gov).

## Safety

### Anakinra

In the large retrospective study by Colafrancesco *et al.*, adverse events (AEs) were reported in 47 of 140 patients. *In situ* (59.5%) or diffuse skin reaction (25.5%) and infections (14.8%; three cases of pneumonia, three cases of urinary tract infections and one case of recurrent dental abscesses) were the main AEs. The majority of affected patients were treated with 100 mg anakinra per day. Severe skin reactions did not resolve during ongoing treatment and led to therapy discontinuation in 75% of the cases. In addition to these AEs, leucopenia (2.1%), thrombopenia (4.2%), and lymphoproliferative disorders (2.1%) were also observed during a mean follow-up period of 35 months. The authors therefore concluded that the frequency of AEs was higher overall than those in previous reports. However, there were no major safety concerns.^8^

Recently, the retrospective single-center cohort study by Campochiaro *et al.* reported injection-site reactions as the most frequent AE, occurring in 4 of 41 participants with one case of zoster infection reactivation. Anakinra was administered at a dose of 100 mg daily in most patients.^13^ In addition, drug-related skin rash was also reported in 1 patient with AOSD in a retrospective study by Dall’Ara *et al.*^16^ involving 18 patients, of whom 13 received anakinra.

The randomized, placebo-controlled study of anakinra in patients with AOSD by Schanberg *et al.*^14^ reported no unexpected safety findings, without further elaboration.

With respect to safety outcomes, treatment withdrawal due to AEs was reported in 16 of 58 patients with AOSD treated with biologic agents in a large retrospective study by Sfriso *et al.*, without further information on the different classes of biologics. However, that study included data that were also included in the study by Colafrancesco *et al.* Therefore, to avoid possible overestimating AEs by counting cases twice, we decided to not fully report the study by Sfriso *et al.*^15^

For the same reason, we will only mention the reported serious adverse events (SAEs) in the study by Vitale *et al.*: a 52-year-old male patient developed pneumonia after 17 months, a 65-year-old male patient developed lower limb ulcers after 110 months, and a 67-year-old male patient developed pneumonia after 9 months of treatment with anakinra. In addition, a 32-year-old female patient died of acute myocarditis at the onset of AOSD only 15 days after the initiation of therapy and a 59-year-old female patient with dilated cardiomyopathy died after 120 months of treatment with anakinra. Overall, the study authors reported AEs and SAEs in 1.7% of patients (n = 72) treated with IL-1 blockers, confirming their good safety profile.^10^

According to the analysis of 27 studies by Vastert *et al.*, injection-site skin reactions are the most common and consistently reported AEs. Importantly, these AEs are mild to moderate in severity and usually resolve within 4 to 6 weeks without the cessation of anakinra. Based on the same review, hepatotoxicity while receiving anakinra is more often observed in patients with prior liver dysfunction.^30^

### Canakinumab

In the phase II, randomized, double-blind, placebo-controlled, multicentre, investigator-initiated trial by Kedor *et al.*, a total of four SAEs were recorded in canakinumab-exposed patients (n = 26) receiving a dose of 4 mg/kg every 4 weeks up to a maximum of 300 mg. A 36-year-old biologics-naive female patient developed non-life threatening transaminitis, which resolved upon the cessation of therapy. Liver biopsy findings led to the diagnosis of drug-induced hepatotoxicity. A 51-year-old female suffered patellofemoral pain syndrome, a 30-year-old male patient developed deep vein thrombosis, and a 66-year-old female patient experienced hypotonia, which led to hospitalization. Furthermore, this trial reported 47 adverse events (AEs) in the verum group, of which 17 were nonserious infections (nasopharyngitis in most cases) and 10 were gastrointestinal disorders (mostly nausea). The authors calculated the exposure times in the canakinumab- and placebo-arms before and after rescue therapy and reassuringly concluded that the AE rate per 100 patient-years of exposure was similar in the two groups.^11^

A retrospective longitudinal outcome study of 50 consecutive patients with refractory AOSD by Laskari *et al.* showed two cases of severe pneumonia, one of which led to treatment cessation. However, neither the exact dosing scheme of canakinumab (ranging from 150 mg every 8 weeks to 300 mg every 4 weeks) nor specific details about previous biologic treatments in these patients were mentioned. In all, 20% of the participants experienced infections: five in the respiratory tract, two in the lower urinary tract, one fungal infection in the oral cavity, one fungal infection in the external genital area, and one mild skin and soft tissue infection caused by Staphylococcus. Drug-related leucopenia occurred in three patients. The authors concluded that canakinumab was safe and well tolerated by most of the patients during the long-term follow-up period of 24 months.^4^

There were a total of three AEs in a single-center observational study by Tomelleri *et al.* within a cohort of 13 patients with AOSD: herpes zoster reactivation, prostatitis, and mild leucopenia. Treatment with canakinumab 4 mg/kg every 4 weeks was only temporarily withheld.^6^

In the report by Campochiaro *et al.*, a 69-year-old male with a systemic form of AOSD was treated with canakinumab 300 mg every 4 weeks following the failure of anakinra and tocilizumab and then developed leucopenia. Another patient in the same cohort, a 51-year-old female with the chronic articular disease type, developed a herpes zoster infection during treatment with canakinumab 300 mg every 4 weeks in combination with methotrexate 20 mg weekly after the failure of anakinra. Both participants were receiving background concomitant treatment with prednisolone ⩾ 10 mg daily. No other AEs occurred in that retrospective study involving 10 patients during follow-up with a median of 9 months.^27^

Ugurlu *et al.* retrospectively collected data on 10 patients with AOSD treated with canakinumab and reported a case of the reactivation of latent tuberculosis 9 months after the first injection with 150 mg while receiving chemoprophylaxis with isoniazid. The authors attributed this outcome, to a certain extent, to prior treatment exposure to multiple biologic agents, without being more specific. Previous treatment with infliximab (n = 3), adalimumab (n = 2), and etanercept (n = 3) was observed in this study.^9^

Patients in the studies by Vitale *et al.*^5^ (n = 9), Cavalli *et al.*^7^ (n = 4), Colafrancesco *et al.*^8^ (n = 4), Kougkas *et al.*^22^ (n = 1) did not experience any AEs during treatment with canakinumab.

### Tadekinig alfa

Gabay *et al.* studied the novel IL-18 blocker tadekinig alfa in patients with refractory AOSD in a phase II, open-label study. The participants were divided into two groups based on the dosing scheme: group one (n = 10) received 80 mg three times weekly, and group two (n = 13) received 160 mg three times weekly. One patient in group two had to stop treatment 1 week after the initiation of the trial due to injection-site reactions. Most of the 47 reported drug-related AEs were skin reactions, mild upper airway infections and arthralgias. A 60-year-old participant developed toxic optic neuropathy, which led to the permanent discontinuation of the study. However, that patient suffered from various cardiovascular comorbidities and had experienced serious thrombotic episodes prior to enrolment in the study. The authors mentioned that the diagnosis of non-drug-related ophthalmic vein thrombosis could not be ruled out. The overall safety profile of tadekinig alfa was good.^28^

In a case report by Kiltz *et al.*, one patient treated with 160 mg tadekinig alfa three times weekly suffered from an upper airway infection. However, the risk of infection in that particular participant was probably already increased due to prolonged exposure to higher doses of corticosteroids.^29^

## Discussion

The introduction of IL-1 inhibitors in the treatment of AOSD has changed the approach to this rare disease considerably. In fact, with this highly effective intervention, we can nowadays control the activity and progress of disease early on. By sparing unnecessary exposure to prolonged high-dose glucocorticoids, we can also avoid, otherwise inescapable, multifold side effects. Recently published studies in sJIA even suggest that first-line treatment with IL-1 inhibitors can substantially increase the proportion of patients reaching drug-free remission on the long-term.^31^ This raises hope of shifting the course of disease to a monocyclic form or even gives a future vision of cure of the disease.

The results of this systematic review indicate that we can expect a fast and robust response in the majority of AOSD cases under IL-1 inhibition. Furthermore, it provides evidence that articular as well as systemic manifestations of AOSD can respond to this approach in the majority of patients. In this context, IL-1 inhibition has shown efficacy in specific organ involvements, for example, of the lungs as well as in other systemic complications of disease, which are named hyper-inflammation, cytokine storm, pre-macrophage activation syndrome (MAS), and MAS.^32^

With the approval of canakinumab and anakinra for treatment of AOSD by European Medicines Agency (EMA) and of canakinumab by the Food and Drug Administration (FDA), we have licensed options that make off-label applications mostly unnecessary. This will allow us to treat especially severe and refractory cases without any delay. Of note, a switch from one to the other IL-1 blocking agent is possible and recommendable if response to treatment is lost or in case of specific adverse events like injection-site reactions.

However, the question of optimal dosing needs clarification in the future. Especially for anakinra, the proposed dose regimen for AOSD is rigid and questionable, since an adjustment of the daily dose according to the individual situation of the patient is often required. In fact, we have learned from canakinumab that the required drug levels in AOSD are usually higher in comparison to the standard application in monogenetic fever syndromes like cryopyrin-associated periodic syndrome (CAPS) or Familial Mediterranean fever (FMF).

**Figure 2.**
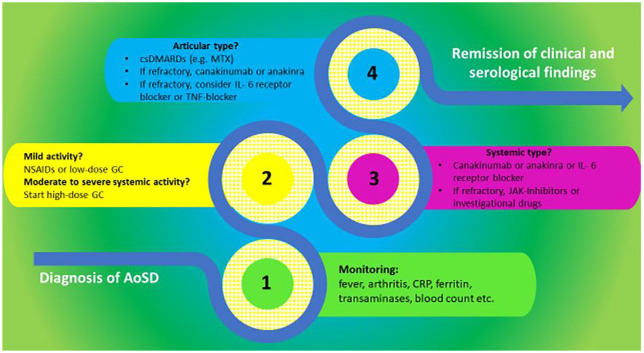
Suggested strategy for management of AOSD. TNF and IL-6 receptor inhibition is presented here as third option as there is no approval for AOSD in USA or Europe. Nevertheless, there is evidence that IL-6 is a practical option for AOSD.^33,34^ AoSD, adult-onset Still’s disease; CRP, C-reactive protein; csDMARDs, conventional synthetic disease-modifying antirheumatic drugs; GC, glucocorticoids; IL, interleukin; JAK, Janus kinase; MTX, methotrexate; NSAIDs, non-steroidal anti-inflammatory drugs; TNF, tumor necrosis factor.

Regarding the safety, the findings in this updated review match those in earlier reports, confirming the high tolerability of IL-1 blockers with no new safety signals identified. The most common AEs, which were mainly mild to moderate in severity, were injection-site reactions, infections, and infestations. Patients with rheumatic diseases, however, have a higher risk of infections. This is not only because of the known immune dysfunction in auto-inflammatory and autoimmune disorders, but also because of the concomitant treatment with high-dose steroids. Although this review is based on a small sample of published data, the findings suggest that the benefits of canakinumab and anakinra clearly outweigh the potential harms or risks. Thus, currently proposed treatment strategies of AOSD already implement IL1-blockers early in the disease course as simplified illustrated in Figure 2. For widely accepted management of AOSD, tools for disease monitoring and international treatment guidelines are currently in development.

There are many potential therapeutic options for the treatment of AOSD. Our focus in this revision was the IL-1 pathway. As shown in Figure 2, tumor necrosis factor and IL-6 inhibition are practical options although there is no approval for its use in AOSD. Castañeda *et al.* and Kaneko *et al.* published a very good revision on Tocilizumab in AOSD.^33,34^

## Conclusion

In summary, with the approval of two IL-1 inhibitors for AOSD we have moved into a new era in the treatment of this rare disease. The overall efficacy-safety profile of both available IL-1 inhibitors is favorable. After a long period of collecting clinical data from cohorts worldwide and with the support of small controlled trials, this first targeted approach in adults with Still’s disease is nowadays a standard of care. We can expect that the successful introduction of these novel therapies will facilitate further clinical and basic research in this field with impact on other auto-inflammatory and hyper-inflammatory conditions.

## Supplemental Material

sj-docx-1-tab-10.1177_1759720X211059598 – Supplemental material for Update on the therapy of adult-onset Still’s disease with a focus on IL-1-inhibition: a systematic review Click here for additional data file. Supplemental material, sj-docx-1-tab-10.1177_1759720X211059598 for Update on the therapy of adult-onset Still’s disease with a focus on IL-1-inhibition: a systematic review by Claudia Kedor, Stylianos Tomaras, Daniel Baeumer and Eugen Feist in Therapeutic Advances in Musculoskeletal Disease

## Notes

1. To avoid potential bias by counting cases twice, we contacted some of the authors via e-mail and asked for clarifications regarding shared data sets of AoSD patients in published case series and cohort studies.
