# Supplementary material for: Update on the therapy of adult-onset Still’s disease with a focus on IL-1-inhibition: a systematic review
Source: Ther Adv Musculoskelet Dis. 2021 Nov 24;13:1759720X211059598. doi: 10.1177/1759720X211059598 (PMC8641116; doi:10.1177/1759720X211059598)
Supplement: sj-docx-1-tab-10.1177_1759720X211059598 – Supplemental material for Update on the therapy of adult-onset Still’s disease with a focus on IL-1-inhibition: a systematic review [file sj-docx-1-tab-10.1177_1759720X211059598.docx]

# Supplementary Material

# Title:

**Update on the therapy of adult-onset Still’s disease with a focus on IL-1-inhibition: A systematic review**

**Literature Search Adult onsets Stills Disease (Therapeutic Advances in Musculoskeletal Disease):**

**Publication Type**: Systematic Review

Update on the use of IL-1 inhibitor´s and other treatments in adult onset stills disease (AOSD).

**Search Terms:**

a) the terms and abbreviations for AOSD

b) the trade and generic names for the three anti-IL-1 agents, Tadekinig and IRAK Inhibitors

Aims: **Canakinumab (Ilaris),** **Anakinra (Kineret), Rilonacept, Tadekinig, IRAK Inhibitors,**

**Timeline:** **2016-2021**

**Database: BIOSIS Preview, Embase databases, Cochrane Library**

1. **Literature Search Terms:**

**Canakinumab:**

| adult onset Stills disease | OR |
| --- | --- |
| adult-onset Stills disease | OR |
| adult onset stills disease | OR |
| adult onset Still disease | OR |
| Still’s Disease Adult-Onset | OR |
| adult onset Still | OR |
| AOSD | OR |
| Canakinumab | AND |
| Ilaris | OR |

**Anakinra:**

| adult onset Stills disease | OR |
| --- | --- |
| adult-onset Stills disease | OR |
| adult onset stills disease | OR |
| adult onset Still disease | OR |
| Stills Disease Adult-Onset | OR |
| adult onset Still | OR |
| AOSD | OR |
| Anakinra | AND |
| Kineret | OR |

**Rilonacept**

| adult onset Stills disease | OR |
| --- | --- |
| adult-onset Stills disease | OR |
| adult onset stills disease | OR |
| adult onset Still disease | OR |
| Still’s Disease Adult-Onset | OR |
| adult onset Still | OR |
| AOSD | OR |
| Rilonacept | AND |
| Arcalyst | OR |

**Tadekinig**

| adult onset Stills disease | OR |
| --- | --- |
| adult-onset Stills disease | OR |
| adult onset stills disease | OR |
| adult onset Still disease | OR |
| Still’s Disease Adult-Onset | OR |
| adult onset Still | OR |
| AOSD | OR |
| Tadekinig | AND |
| interleukin 18 binding protein | OR |

**IRAK inhibitors (check if there is anything published in AOSD or other rheumatologic diseases):**

| adult onset Stills disease | OR |
| --- | --- |
| adult-onset Stills disease | OR |
| adult onset stills disease | OR |
| adult onset Still disease | OR |
| Stills Disease Adult-Onset | OR |
| adult onset Still | OR |
| AOSD | OR |
| IRAK inhibitor | AND |
| IRAK inhibitor | OR |

- **Irak inhibitors were excluded due to lack of published data.**

### **PRISMA flow diagram of record selection process: Canakinumab, Anakinra, Rilonacept, Embase, Biosis Abstracts, Cochrane (08.03.2021)**


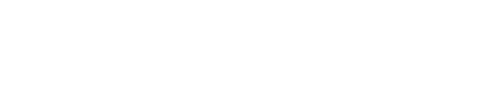


Records identified through database searching

(n = 1013)


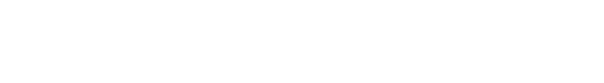


Records after duplicates removed (n =986)


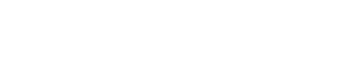

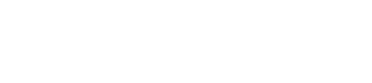


Records screened (n =986)

Records excluded (n =885)


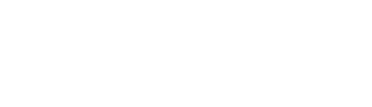

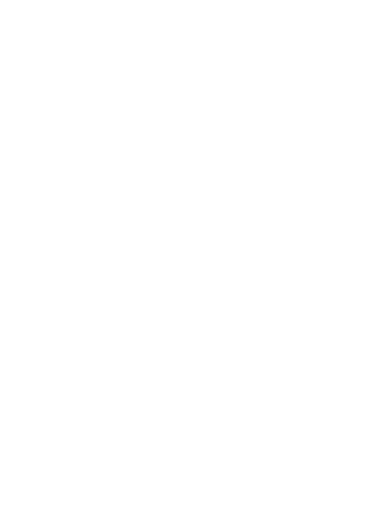


Full-text documents assessed for eligibility (n =121)

Full-text documents excluded (n = 104 total)

Abstracts with full publication or review publication (n=4)

Excluded, irrelevant or incomplete data set (n=100)

Eligible (n=17)

**Table 1: Overview on IL-1 Inhibitors in AOSD, outcome and experience on steroid sparing**

| **Primary Author** | **Year** | **Drug** | **Study**  **Design** | **Patient Number** | **Systemic** | **Arthritic** | **Duration of Treatment** | **Degree of Remission** | | | **Steroid Use** | | |
| --- | --- | --- | --- | --- | --- | --- | --- | --- | --- | --- | --- | --- | --- |
|  |  |  |  | **n** | **AOSD^1^** | **AOSD** | **(month)** |  |  |  |  |  |  |
|  |  |  |  |  |  |  |  | **Complete** | **Partial** | **None** | **n** | **Stopped** | **Reduced** |
| Laskari K. et al. | 2020 | CAN | M,R | 50 |  | 67% | 12 | 68% | 16% | 16% | 41 | 21 (weaned) | n.a. |
| Vitale A. et al. | 2020 | CAN | M,R | 9 | 7 | 2 | 15 ± 12.3 | 8 | n.a. | 1 | n.a. | n.a. | n.a. |
| ^1^Tomerelli | 2020 | CAN | SC,R | 13 | n.a. | n.a. | 3-18 | 13 | 0 | 0 | 13 | 3 | 10 |
| Cavalli, G. et al. | 2019 | CAN | R | 4 | 4 | **n.a.** | n.a. | 4 | n.a. | n.a. | 4 | 2 | n.a. |
| Colafrancesco, S., et al. | 2017 | CAN | R | 4 | 3 | 1 | 22.1 ± 6.5 | 3 | 0 | 1 | 4 | 0 | 3 |
| ^1^Urgurlu et al. | 2018 | CAN | R | 10 | n.a. | n.a. | 43 ± 33 | 10 | 0 | 0 | 10 | 4 | n.a. |
| Vitale A. et al. | 2016 | CAN | R | 3 | n.a. | n.a. | n.a. | 2/3(66.66%) | 1/3(33.33%) | 0/3  (0%) |  |  |  |
| Kedor et al. 2020 | 2020 | CAN | RCT | 18 | 0 | **18** | 24 | 10 | 8 | 0 | stable dose | ≤10 mg/day prednisolone | |
| Bodard et al. | 2021 | ANA | M,R | 96 | n.a. | 24 (25%) | n.a. | n.a. | n.a. | n.a. | n.a. | n.a. | n.a. |
| Campochiaro et al. | 2021 | ANA | SC, R | 41 | 25 (61%) | 16 (39%) | 24 | 20 (29%) | 24% | 3 (7%) | 41 (23 ±18 Prednisone) | 38%(EOT) | n.a. |
| Schanberg et al. | 2020 | ANA | RCT | 12 | 3 | 0 | 0.5 | 3 | 0 | 0 | n.a. | n.a. | n.a |
| Colafranchesco et al. | 2017 | ANA | M,R | 140 | 104 (74,2 %) | 36 (25,8%) | 35.7 36.1 | 20 (28,1%) | n.a. | n.a. | 97% (Bsl)31.8% (22/69) | 43,4% after 12 month | n.a. |
| Sfriso et al. | 2016 | ANA | M,R | 34 | n.a. |  | n.a. | 26 | 7 | 1 | 100 | n.a. | n.a. |
| Vitale et al. | 2016 | ANA | M,R | 78 | n.a. |  | n.a. | 61/78(78.20%) | 10/78(12.82%) | 7/78  (8.97%) | n.a. | n.a. | n.a. |
| Vitale et al. | 2020 | ANA | M,R | 141 | 105 | 36 | 12 | n.a. | n.a. | n.a. | n.a. | n.a. | n.a. |
| Dall`Ara | 2016 | ANA | M,R | 13 | 8 | 5 | 12-102 | 13 | 1 | n.a. | 6 | n.a. | n.a. |
| Petryna & Gao | 2016 | RIL | R, SC | 2 | n.a. | n.a. | 3 | Good response | n.a. | n.a. | n.a. | n.a. | n.a. |

M multicenter, R retrospective, SC single center, RCT, randomized controlled trial, ^1^ Abstract
